# Data-Driven External-Load Analytics: Integrating Cluster Analysis and ACWR Monitoring in Elite Handball

**DOI:** 10.5114/jhk/217125

**Published:** 2026-04-02

**Authors:** Nebahat Eler, Tevfik Cem Akalın, Serdar Eler, Marko Joksimovic

**Affiliations:** 1 Faculty of Sport Sciences, Gazi University, Ankara, Turkiye.; 2 Faculty of Sport Sciences, Zonguldak Bulent Ecevit University, Zonguldak, Turkiye.; 3 Faculty for Sport and Physical Education, University of Montenegro, Nikšić, Montenegro.

**Keywords:** handball, cluster analysis, ACWR, external load, wearable sensors

## Abstract

The aim of this study was to characterize multidimensional external-load profiles obtained from sensor-based tracking data over a four-month competitive period in an elite men’s handball team and to investigate their associations with the session type, the playing position, and weekly workload fluctuations, as measured by the acute:chronic workload ratio (ACWR). Data were collected from 23 elite players using an ultra-wideband tracking system. Six variables, i.e., total distance, maximum speed, accumulated acceleration load (AAL), the number of exertions, maximum jump height, and the number of jumps ≥0.30 m, were standardized and clustered using k-means (k = 4). The cluster with the highest composite z-score was defined as a high load. The association between weekly density of high-load sessions and the likelihood of an ACWR spike (≥1.30) was tested using logistic regression. Results showed that match sessions were 1.8 times more likely than training sessions to fall into the high-load cluster. Wings and center-backs were significantly more represented in high-load clusters than goalkeepers and pivots. Additionally, when 15% or more of the previous week’s sessions were classified as of the high load, the odds of an ACWR spike in the following week increased by 10.7 times. These findings suggest that data-driven (unsupervised) clustering of external-load variables supports early identification of high-risk workload patterns. Monitoring the weekly distribution of high-load sessions may help mitigate fatigue-related maladaptation by enabling proactive, position-specific load management in elite handball.

## Introduction

Monitoring and managing training loads have become pivotal practices in elite team sports, essential for both performance optimization and injury prevention. Handball is a high-contact, intermittent sport that presents a highly complex external load profile, characterized by repeated accelerations, decelerations, jumps, and collisions. This complexity makes it necessary to use multidimensional assessment strategies to evaluate sport-specific demands (Carton-Llorente et al., 2023). Recent advances in wearable-sensor technology and sensor-based tracking systems now allow the external load to be measured with high temporal and spatial resolution, fostering the development of individualized, position-specific training strategies (Font et al., 2021).

One of the primary challenges in load monitoring is not limited to the quantitative evaluation of physical output, but rather lies in the accurate interpretation of its temporal dynamics, particularly the fluctuations between acute and chronic workloads. The acute:chronic workload ratio (ACWR) has become a widely used metric for predicting injury risk on the basis of short- and long-term load data; numerous studies have shown that ACWR values exceeding 1.30 elevate injury risk across several sports, from football to volleyball (Bowen et al., 2020; Schumann et al., 2023). However, the application of such analyses in handball remains limited, and studies integrating data-driven multivariate load profiling are rare.

External measures such as total distance, the acceleration load and high-speed running exhibit strong correlations with internal-load indicators under complex, high-intensity demands (McLaren et al., 2018). Yet these correlations can vary by sport, the game model, the playing position and the recovery protocol (Maciel et al., 2016; Møller et al., 2017). Worst-case scenarios (WCSs), defined as brief periods of the maximal physical demand, vary significantly across playing positions and often exceed the average match load (Carton-Llorente et al., 2023). This highlights the limitations of relying solely on mean values when planning training or match strategies.

Recently, unsupervised machine-learning algorithms such as k-means clustering have been adopted in sports science to classify training and match sessions according to multivariate load profiles, enabling the identification of natural “high-load clusters” that elude traditional threshold-based analyses (Bautista et al., 2016). Although such data-driven approaches are increasingly common in football and basketball (Bautista et al., 2016; McLaren et al., 2018), their use in handball is still scarce. Carton-Llorente et al. (2023) characterized position-specific loads via WCS analysis, yet did not employ multivariate clustering; Font et al. (2021) likewise described positional load profiles without data-driven cluster classification. Møller et al. (2017) reported that >60 % weekly increases in training and match volume doubled shoulder-injury risk in young elite handball players, but relied on simple load-change ratios rather than multivariate load profiling. To address this gap, we present a data-driven external-load analytics approach that integrates unsupervised cluster analysis with non-overlap ACWR monitoring to describe and predict workload patterns in elite men’s handball.

The aim of this study was to characterize the multidimensional external load profiles derived from sensor-based tracking data over a four-month competitive period in an elite men’s handball team and to investigate their associations with the session type, the playing position, and weekly load fluctuations, as measured by the acute:chronic workload ratio (ACWR). The following hypotheses were tested:

H1. Match sessions are statistically more likely to fall into the “high-load” cluster than training sessions.

H2a. Wings enter the high-load cluster more frequently than goalkeepers and pivots.

H2b. Centre-backs enter the high-load cluster more frequently than lower-load positions such as pivots and goalkeepers.

H3. Weeks in which the ACWR ≥1.30 are preceded by significantly higher proportions of high-load sessions than weeks with the ACWR <1.30.

By combining k-means clustering with weekly ACWR calculations, the study aimed to provide practitioners with actionable insights for position-specific load management and identification of team-level workload imbalance patterns that may warrant closer monitoring, thereby facilitating data-driven individualization of training plans and the development of injury-prevention strategies.

## Methods

### Participants

This study employed a prospective, observational, longitudinal design spanning a four-month period (first half of the season) from 1 October 2022 to 24 January 2023. A total of 23 elite male handball players from a single club competing in the Turkish Super League voluntarily participated in the study (age = 24.3 ± 4.1 years; body height = 191.2 ± 7.5 cm; body mass = 93.6 ± 9.8 kg) (Table 1). The squad consisted of eight center backs, five wings, five pivots, and five goalkeepers.

**Table 1:** Demographic characteristics of the participants (n = 23).

Variable	Mean ± SD	Min–Max
Age (years)	24.3 ± 4.1	19–32
Stature (cm)	191.2 ± 7.5	178–205
Body mass (kg)	93.6 ± 9.8	78–110
BMI (kg·m⁻^2^)	25.6 ± 1.8	22.9–29.8

The inclusion criteria were as follows: (i) a minimum of three years of professional playing experience; (ii) no history of major injury within the previous three months; (iii) participation in at least 65% of all team training sessions or matches during the observation period, and (iv) belonging to the first-team squad of the club competing at the same competitive level during the observation period.

The exclusion criteria were as follows: (i) player or player-session observations with more than 50% missing data in any external-load variable; and (ii) player-session observations that failed to yield valid external-load measurements due to technical issues (e.g., sensor malfunction or recording interruptions) during data cleaning. External-load data were structured at the player-session level, such that each row represented the data of a single athlete from a specific team event (a training session or a match). In total, 721 player-session observations were recorded (training: n = 618; match: n = 103). The median number of athletes contributing data per team event was 18 (range = 14–23). For the primary analyses, complete player-session observations were retained (n = 695; 96.4%) (Table 3).

**Table 2:** Descriptive statistics for six external-load variables (mean ± SD) across all sessions and by the session type (training vs. match).

Variable	All Sessions Mean ± SD	TrainingMean ± SD	MatchMean ± SD
Distance (m)	3677.19 ± 1360.18	3678.32 ± 1292.88	3670.66 ± 1703.74
Max Speed (km·h⁻^1^)	22.16 ± 4.43	21.93 ± 4.49	23.47 ± 3.78
Accumulated Acceleration Load (AU)	490.55 ± 154.14	495.38 ± 147.41	462.74 ± 186.61
Exertions (count)	20.69 ± 33.85	18.91 ± 30.92	30.90 ± 46.15
Max Jump Height (cm)	16.95 ± 21.56	18.49 ± 22.64	8.08 ± 10.02
Jumps ≥0.30 m (count)	3.83 ± 8.61	3.86 ± 8.83	3.65 ± 7.21

**Table 3:** Distribution of high-load-cluster membership by the session type.

Session type	High load, n (%)†	Other, n	Total, n
Match	24 (23.3 %)	79	103
Training	89 (14.4 %)	529	618

† Percentage = (High-load ÷ Total) × 100

The study was conducted as a retrospective analysis of pre-existing training and match data routinely collected by the club’s performance department for monitoring purposes. Ethics approval was obtained from the Ethics Committee of the Gazi University, Ankara, Turkey (protocol code: 2025-1568; approval date: 22 September 2025) to permit the research use of these anonymized data, and all procedures were conducted in accordance with the Declaration of Helsinki. All athletes had previously provided written informed consent for their anonymized data to be used for research purposes.

### Design and Procedures

This study employed a prospective, observational, longitudinal design spanning a four-month period (first half-season) from 1 October 2022 to 24 January 2023. All training sessions and matches of the first-team squad from a single elite handball club were monitored, yielding a total of 721 player-session observations (training = 618; matches = 103). A player-session observation represents the external-load data of a single athlete recorded during a given team training session or a match; thus, multiple athletes contribute observations to the same team event, and in some cases an athlete may contribute more than one record within the same calendar day due to split sessions or segmented data exports.

**Figure 1:**
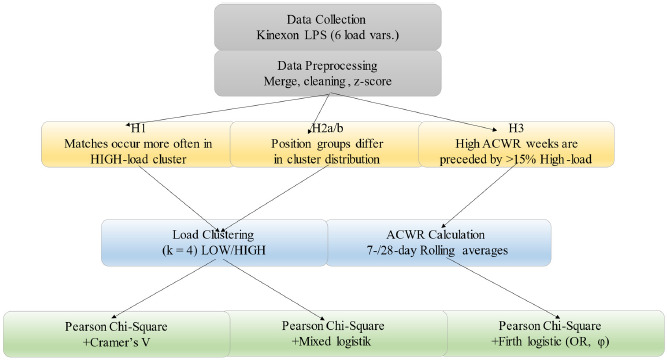
Study design.

The median number of athletes contributing data per team event was 18 (range = 14–23). After applying the predefined missing-data rules, complete player-session observations were retained for the primary analyses (n = 695; 96.4% of all player-session observations; Table 3). Figure 1 illustrates the prospective observational design and the flow of player-session observations across the four-month period.

#### Unit of Analysis and the Level of Inference

In the present study, the primary unit of analysis for session-level comparisons was the player-session observation, defined as the external-load data of a single athlete recorded during a given team training session or a match. This structure reflects standard practice in team-sport load monitoring, where multiple player-level observations are nested within the same team event.

Importantly, all inferential interpretations were restricted to the team level. Weekly ACWR values and subsequent spike analyses were computed using aggregated team-level data, and no individual-level injury risk inference was made. Accordingly, the analyses aimed to describe workload patterns and associations, rather than to establish causal or player-specific risk prediction. This distinction underpinned the analytical framework and ensured conceptual consistency and replicability.

### Measures

The external performance load was recorded with an ultra-wideband (UWB) local positioning system (Kinexon LPS, Kinexon GmbH, Munich, Germany) sampling at 20 Hz. Each athlete wore a uniquely coded ultra wide-band (UWB) transmitter fixed at the sternum level by means of an elastic chest strap. The indoor court was calibrated with eight fixed antennas, providing <10 cm positional accuracy in all three axes (Kinexon, 2025) At the end of every session, raw data were exported as .csv files and processed in Python 3.11 (pandas 2.2.2) to obtain the external-load variables listed below. We did not have concurrent internal-load or wellbeing measures (e.g., session-RPE, heart-rate derived indices, HRV, or wellness questionnaires) available in this retrospective dataset; therefore, the present analyses focused on external-load indicators captured by the local positioning system.

Elite handball load monitoring is advised to address at least three dimensions: (i) volume (total distance), (ii) mechanical stress (accumulated acceleration load [AAL], exertions), and (iii) intensity/neuromuscular demand (maximum speed, vertical load). Exertions were defined as discrete high-intensity acceleration or deceleration events exceeding manufacturer-defined inertial thresholds, reflecting repeated explosive mechanical efforts independent of locomotor speed. Linke et al. (2018) reported that total distance reliably reflected in-season load fluctuations, whereas the model of Windt and Gabbett (2019) showed increased injury risk when chronic readiness failed to rise during high-volume weeks. Since handball involves frequent high-intensity accelerations and decelerations, acceleration-based metrics such as AAL are indispensable beyond distance-based measures. A sustained reduction in weekly maximum-speed exposure can impair sprint capacity and hamstring health (Duhig et al., 2016; Malone et al., 2017). Moreover, the typical “worst-case scenario” profile of wing players is characterized by high vertical loads (Hoppe et al., 2018), rendering jump height and ≥0.30 m jump critical neuromuscular indicators. The apparent unit difference reflects the distinction between a continuous outcome variable (maximum jump height) and a threshold-based count variable (jumps ≥0.30 m). No inconsistency in measurement units is present. Accordingly, the present study adopted a six variable, balanced monitoring framework spanning volume, mechanical and neuromuscular axes.

#### Data Processing and Derived Variables

All procedures were implemented in a reproducible Python-based pipeline using pandas, scikit-learn, and matplotlib, ensuring clear and transparent data processing

#### Participant Characteristics and Anthropometric Measurements

All anthropometric data were collected during the first week of the measurement period (September 2022) in the club’s sports-medicine laboratory. Measurements were taken by a professional physiotherapist and a strength-and-conditioning coach in accordance with the International Biological Program (IBP) protocol. The stature (cm) was measured barefoot with a stadiometer (SECA 213, Hamburg, Germany; ±0.1 cm) with the head in the Frankfurt plane.

Body mass (kg) was recorded on a digital scale (Tanita BC-418, Tokyo, Japan; ± 0.1 kg) in a fasted state prior to training. The body mass index (BMI, kg · m⁻^2^) was calculated as mass / height^2^. Age (years) was computed automatically from official license records. The playing position was categorized by coaching staff before the season as a wing, a centre-back, a pivot or a goalkeeper.

Data are reported as n (%) unless otherwise indicated. All measurements were taken in a single session; the stadiometer and scale were recalibrated after every 10 athletes.

#### Raw-Data Cleaning

From the Kinexon files, six external-load indicators were extracted: total distance (m), maximum speed (km·h⁻^1^), accumulated acceleration load (AAL, AU), exertions (count), maximum jump height (cm) and jumps ≥0.30 m (count) (Table 2). A regular-expression parser split the date string to create the session type (MATCH/TRAIN) and date columns. Any player × variable combination with >50% missing cells was removed, following methodological guidelines on bias and statistical-power loss due to missing data (Davids et al., 2006; Schulz and Grimes, 2002). For the remaining indicators, the overall missing rate was <3%; these cases were handled by listwise deletion.

#### Load Clustering (High-Load Cluster)

The six external load variables, including total distance, maximum speed, accumulated acceleration load (AAL), the number of exertions, maximum jump height, and the number of jumps equal to or exceeding 0.30 m, were standardized using z-scores. A k-means clustering algorithm (scikit learn; k = 4, n<sub>init</sub> = 10, random state = 42) was then applied. Because external-load indicators such as distance, peak speed, cumulative acceleration and vertical load are typically highly inter-correlated, evaluating each metric against isolated thresholds may fail to reflect the true mechanical-metabolic cost of a training or a match session. Consequently, a multivariate approach was adopted instead of analyzing single variables in isolation (Gómez-Carmona et al., 2022). For each cluster, the mean composite z-score was calculated. The cluster with the highest average score was designated as the high load cluster. A binary variable coded as 0 or 1, referred to as HighLoad, was added to the dataset and used as the dependent variable in hypothesis testing for H1 (match versus training) and H2a and H2b (comparisons by the playing position).

#### Acute:Chronic Workload Ratio (ACWR)

The Acute:Chronic Workload Ratio (ACWR) was derived from team-level AAL data to quantify weekly load fluctuations. The chronic load was calculated as the rolling mean of the previous four weeks, excluding the current week (non-overlap model). First, a daily load was obtained for each calendar day (daily team load = Σ<sub>i=1…N</sub> AAL<sub>i,t</sub>). The acute load (Acute<sub>t</sub>) was the sum of the most recent seven days, whereas the chronic load (Chronic<sub>t</sub>) was the 28-day mean.

The ACWR was then calculated as Acute<sub>t</sub> / Chronic<sub>t</sub>. Ratios were resampled to ISO weeks (Monday ending), assigning one ACWR value per week. Weeks with the ACWR ≥1.30 were tagged as an ACWR spike (1 = spike, 0 = normal). The predictor HighLoad PrevWeek was coded 1 when the proportion of high-load sessions in the previous week exceeded the median threshold (≥15%); otherwise 0. This dichotomous variable was used in firth-corrected logistic models and chi square analyses to predict ACWR spikes. The approach enabled week-by-week monitoring of how short-term (acute) load increases exceeded longer-term (chronic) preparedness, with the ACWR ≥1.30 serving as a clinically meaningful risk flag.

### Statistical Analysis

Raw Kinexon files were merged in Python 3.11/Pandas 2.2.2. The six external-load metrics (total distance, maximum speed, AAL, exertions, maximum jump height, ≥0.30 m jumps) were z-scaled, because the overall missing-cell rate was <2%, listwise deletion was applied (Hopkins et al., 2009).

*High-load cluster definition*. Standardized variables were clustered with k-means (k = 4, 10 random starts, 300 iterations) (MacQueen, 1967). Candidate solutions (k = 3–6) were evaluated using average silhouette scores (Rousseeuw, 1987) (Table S1), which indicated weak-to-moderate separation. Although k = 3 yielded a slightly higher silhouette score than k = 4, we retained k = 4 as the primary solution to preserve interpretability and to maintain a distinct high-load cluster while providing additional granularity in the remaining load profiles. Cluster stability was checked in sensitivity analyses with k = 3 and k = 5. Sensitivity analyses with k = 3 and k = 5 yielded qualitatively similar position-specific patterns; therefore, k = 4 was retained as the primary clustering solution. The cluster of which centroid had the highest mean z-score was designated “high load”.

*Hypothesis 1 (match vs. training)*. Membership in the high-load cluster by the session type was cross tabulated (2 × 2) and evaluated with the Pearson’s chi-square test (χ^2^, df = 1) (Bewick et al., 2005). Effect size φ, odds ratio (OR) and the 95% confidence interval (CI) were reported with Haldane-Anscombe continuity correction (Anscombe, 1973).

*Hypothesis 2 (playing position)*. A 4 × 2 table (wing, centre-back, pivot, goalkeeper × high-load membership) was analyzed with χ^2^ (df = 3). Post-hoc pairwise comparisons used Bonferroni-adjusted χ^2^ tests (Dunn, 1961). A mixed-effects logistic model including player ID as a random intercept provided additional confirmation (Bates et al., 2015).

*Hypothesis 3 (weekly ACWR spikes)*. Daily AAL was summarized into acute and chronic loads as described; the ACWR ≥1.30 defined a spike (Gabbett and Whiteley, 2017). In a sensitivity analysis, the ACWR was also computed using an overlap model in which the chronic window included the current week; results are reported in Table S2. The proportion of high-load sessions in the preceding week was dichotomized at the median threshold (≥ 15%) (Field, 2024). The association was tested with χ^2^ (df = 1), reporting φ, OR and the 95% CI; consistency was verified with Firth-corrected logistic regression to account for small cell frequencies (Firth, 1993). To address temporal ambiguity and potential match-schedule confounding, we conducted two supplementary checks: (i) within weeks classified as HighLoad PrevWeek = 1, we quantified the share of high-load sessions attributable to matches versus training; and (ii) we tested whether match presence in week t (0/1) predicted ACWR spikes in week t+1 independently of high-load-session density by fitting an additional model: Spike(t+1) ~ HighLoadPrevWeek(t) + MatchPresence(t), using Firth correction given the limited number of weeks.

Normality of continuous variables was assessed with the Shapiro-Wilk test, and homogeneity of variance with the Levene’s test. When assumptions were violated, the Mann-Whitney U or the Kruskal-Wallis test was applied (Field, 2024). All tests were two-tailed with α = 0.05.

*Software and reporting*. Analyses were conducted with NumPy, SciPy, scikit-learn and Stats models; visualizations were produced with Matplotlib. Findings are presented in accordance with CONSORT-TL guidelines. Mixed-effects models were used as sensitivity analyses to verify that the observed associations were robust to within-player and within-week dependence structures.

## Results

Tables 3 and 4 show that match sessions were significantly more likely to fall into the high load cluster (χ^2^(1) = 4.64, *p* = 0.031; φ = 0.08) compared to training sessions. The calculated odds ratio was 1.81 (95% CI = 1.09–3.00), indicating that the probability of experiencing high metabolic-mechanical stress was approximately 1.8 times greater in matches than in training. This finding underscores the need for recovery strategies that are specifically tailored to the post-match period throughout the competitive season.

**Table 4:** Association between the session type and membership in the high-load cluster.

Statistical Metric	Estimated Value
Chi-square (df = 1)	4.64
*p* value	0.031*
Effect size (φ)	0.08
Odds ratio (Match – Training)	1.81
95% CI (lower / upper)	1.09 / 3.00

* p < 0.05 (two-tailed)

As shown in Table 5, wings and center-backs entered the high-load cluster significantly more often than goalkeepers and pivots (χ^2^(3) = 13.87, *p* = 0.003, φ = 0.14). Bonferroni-adjusted pairwise tests revealed the greatest difference between wings and goalkeepers (OR ≈ 10), indicating that external-load demands on wing players were roughly ten times higher than those on goalkeepers. These findings support the need for position-specific recovery and load-management strategies.

**Table 5:** Distribution of the high-load-cluster membership by the playing position (six-variable cluster definition).

Position	High load, n (%)†	Other, n	Total, n
Goalkeeper	2 (2.7%)	71	73
Wing	37 (22.0%)	131	168
Centre-back	53 (16.3%)	273	326
Pivot	21 (17.1%)	102	123

† Percentage = (High-load ÷ Total) × 100

Table 5 presents the raw distribution, whereas Table 6 summarizes the 4 × 2 contingency analysis. The chi-square test (χ^2^(3) = 13.87, *p* = 0.003) revealed a significant positional effect on membership in the high-load cluster. The effect size was small-to-moderate (φ = 0.14). The most pronounced pairwise contrast occurred between wings and goalkeepers: an odds ratio of 10.03 (95% CI = 2.35–42.83) indicates that wing players were roughly ten times more likely to fall into the high-load cluster than goalkeepers.

**Table 6:** Association between the playing position and membership in the high-load cluster.

Statistical Metric	Estimated Value
Chi-square (df = 3)	13.87
*p*-value	0.003*
Effect size (φ)	0.14
Odds ratio (Wing vs Goalkeeper)	10.03
95% CI (lower / upper)	2.35 / 42.83

* p < 0.05 (two-tailed)

Pair-wise post-hoc tests (Table 7) showed significant differences only for the Wing-vs-Goalkeeper, Centre-back-vs-Goalkeeper and Pivot-vs-Goalkeeper pairs (Bonferroni-adjusted *p* < 0.0083). Wings were ≈ 10 times, center-backs ≈ 7 times and pivots ≈ 7 times more likely than goalkeepers to enter the high-load cluster. No significant differences emerged among wings, centre-backs and pivots. This pattern confirms that speed- and acceleration-based mechanical stress is concentrated in the more mobile positions, whereas goalkeepers experience markedly lower worst-case external-load exposure (Hoppe et al., 2018).

**Table 7:** Pair-wise comparisons between playing positions (Bonferroni-adjusted).

Comparison (row- column)	χ^2^ (df = 1)	Adjusted *p*	Odds ratio	95 % CI
Wing vs. Goalkeeper	14.06	0.001*	10.03	2.35–42.83
Centre-back vs. Goalkeeper	10.01	0.002*	6.97	2.01–24.18
Pivot vs. Goalkeeper	8.49	0.004*	7.12	1.79–28.31
Wing vs. Centre-back	2.11	0.420	1.36	0.83–2.23
Wing vs. Pivot	1.36	0.600	1.28	0.66–2.48
Centre-back vs. Pivot	0.12	1.000	0.94	0.56–1.57

* p < 0.05

**Figure 2:**
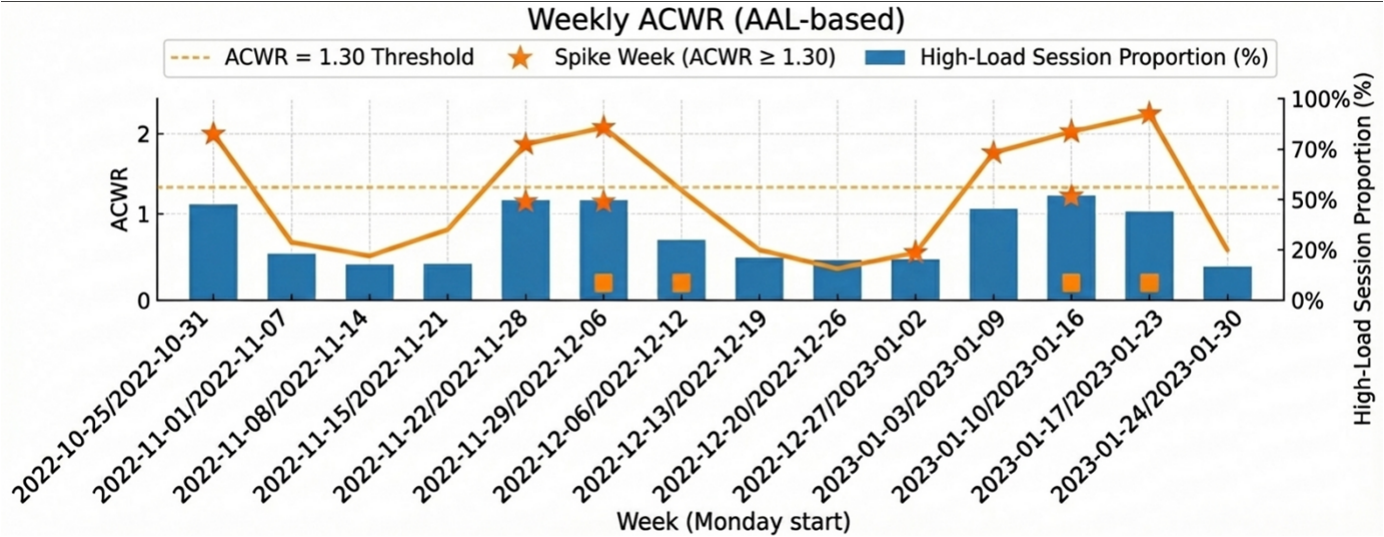
Weekly ACWR.

Examination of Tables 8 and 9 indicates that weeks classified as the ACWR ≥1.30 “spikes” were preceded by a proportionally larger share of high-load sessions (χ^2^(1) = 3.88, *p* = 0.049, φ = 0.49). Weeks in which the high-load-session ratio exceeded the median threshold (≥15%) increased the odds of breaching the ACWR cut-off in the following week by approximately 10.7-fold (95% CI = 1.02–111.20) (Figure 2). However, inference was constrained by the small week-level sample (n = 16), with only 7 weeks meeting the high-load threshold and 5 ACWR-spike weeks, resulting in sparse contingency-table cells (as low as n = 1–3) and a wide confidence interval. Accordingly, the estimate should be interpreted as preliminary, hypothesis-generating associative evidence consistent with H3 rather than a causal or precise predictive effect. From an applied perspective, the pattern suggests that weeks with a higher density of high-load sessions may warrant closer monitoring when planning the subsequent micro-cycle.

**Table 8:** Distribution of ACWR ≥1.30 “spike” weeks according to the previous week’s high-load-session density.

Previous-week high-load-session density*	ACWR spike week (≥1.30), *n*	Other weeks (<1.30), *n*	Total weeks, *n*
High (≥ median, ≥15%)	4	3	7
Low (< median, <15%)	1	8	9

* Median threshold = 0.15; a week was classified as High when ≥15% of its sessions belonged to the high-load cluster

**Table 9:** Association between previous-week high-load-session density and occurrence of ACWR ≥1.30 spikes.

Statistic	Value
Chi-square (df = 1)	3.88
*p*-value	0.049*
Effect size (φ)	0.49
Odds ratio (High vs. Low)	10.67
95% CI (lower/upper)	1.02/111.20

* p < 0.05 (two-tailed)

In supplementary analyses, we further examined whether the observed association could be explained by match scheduling. When match presence in week t was included alongside HighLoadPrevWeek, the association between HighLoadPrevWeek and Spike(t+1) was attenuated but remained directionally consistent, whereas match presence did not show an independent association with ACWR spikes. These findings suggest that the week-to-week association may partly reflect scheduling-related effects and should be interpreted within the constraints of this observational design.

## Discussion

Using a data-driven external-load analytics workflow (unsupervised cluster analysis + non-overlap ACWR monitoring), this study mapped multivariate workload profiles in elite male handball and found statistical support for all three research hypotheses. Below, the findings are discussed in relation to previous works, the methodological choices are interpreted, and practical implications are highlighted.

Six workload variables were analyzed: total distance (cm), maximum speed (km/h), accumulated acceleration load (AAL, arbitrary units), the number of exertions (count), maximum jump height (cm), and the number of jumps equal to or exceeding 0.30 m (count). A single variable alone cannot reflect the full scope of physical demands. Combining variables like AAL, sprint speed, and jump height allows for a more complete understanding of training loads (Maciel et al., 2022). Consequently, the load-injury relationship could be assessed not only in terms of volume but also of the type and intensity.

The lower maximum jump heights observed during matches likely reflect the contextual and biomechanical constraints of competitive play rather than reduced neuromuscular demand. Match jumps are predominantly reactive, contact-limited, and rarely executed under optimal countermovement conditions, whereas training sessions often include structured plyometric drills that allow athletes to reach higher maximal vertical displacement. Accordingly, match play may involve frequent jump actions with lower peak height, while training facilitates higher maximal jump outcomes under controlled conditions.

### Match-Versus-Training Differences

Hypothesis 1 was confirmed: over the season, matches fell into the high-load cluster 1.8 times more frequently than training sessions (odds ratio = 1.81, *p* = 0.031). Thus, matches impose higher physiological and mechanical demands not only on average but also in their worst-case scenarios (WCSs) (Gabbett and Whiteley, 2017). Holm and Randers (2025) also found that match days included significant time spent in high-acceleration zones, even when the overall external load was moderate. Carton-Llorente et al. (2023) showed that positional load differences peaked during the most intense passages of play and were poorly captured by traditional averages. The micro-cycle pattern reported by Holm and Randers (2025), which is characterized by elevated external loads on MD+2 and MD−3 or earlier, followed by a systematic taper as match day approaches, closely reflects the load distribution trend observed in the present study.

### Position-Specific Load Exposure

Hypothesis 2 predicted more frequent high-load membership for wing and center-back players than for goalkeepers and pivots. Wings were ≈ 10 times and center-backs ≈ 7 times more likely than goalkeepers to experience a high-load session (*p* < 0.005). These results match earlier findings indicating that wings and center-backs face higher external loads due to their speed and frequent accelerations (Carton-Llorente et al., 2023; Hoppe et al., 2018). In addition to locomotor demands, wing players’ involvement as second-line attackers in organized offense requires repeated high-intensity off-ball movements and action sequences, which may help explain their higher exposure to high-load clusters (Hatzimanouil et al., 2024). Font et al. (2021) observed significantly higher meters per minute and high-speed-running distances for wings, while center-backs performed the most high-intensity accelerations and decelerations. Contrary to initial expectations, pivots also entered the high-load cluster ≈ 7 times more often than goalkeepers, revealing a hidden neuromuscular burden linked to frequent blocking and pushing actions. Although wings, center-backs and pivots did not differ significantly from one another, each exhibited markedly greater load exposure than goalkeepers (Karcher and Buchheit, 2014). Thus, position-specific load-management protocols are essential for both performance enhancement and injury reduction (Bautista et al., 2016).

### Weekly ACWR Spikes and Accumulated High-Load Sessions

Hypothesis 3 proposed that weeks with the ACWR ≥1.30 would be preceded by a higher proportion of high-load sessions. Indeed, immediately before ACWR-spike weeks, a greater share of sessions belonged to the high-load cluster (φ = 0.49, *p* = 0.049). Quantitatively, when ≥15% of sessions in week t consisted of high-load work, the odds of an ACWR spike in week t + 1 increased by ≈ 10 fold. This finding supports the notion that abrupt acute-load surges impose load disproportionate to chronic preparedness and elevate injury risk (Blanch and Gabbett, 2016; Bowen et al., 2020; Windt and Gabbet, 2019). The 1.30 threshold used here corresponds to the “red-flag” cutoff recommended by Gabbett and Whiteley (2017) and may serve as a pragmatic load-based flag in handball; however, its validity as an injury-risk threshold requires prospective confirmation within handball-specific cohorts.

Lopez-Valenciano et al. (2018) showed that both very high (≥1.54) and very low (<0.76) ACWR values increased injury risk, emphasizing that under-loading can be as hazardous as over-loading. Our findings also suggest that a high number of intense sessions can upset the balance between training loads and preparedness, increasing physiological risk. Integrating subjective feedback (e.g., wellness scores) into individual monitoring systems could strengthen early-warning mechanisms. Distributed Lag Non-Linear Models (DLNM) have shown that the accumulated load better predicts health problems than single-day peaks (Bache-Mathiesen et al., 2022); our observation that high-load density in week t predicts ACWR spikes in week t + 1 concurs with this temporal-accumulation model.

Importantly, ACWR spikes in the present study were used as a load-based risk proxy rather than a clinical endpoint, because injury outcomes were not prospectively tracked. The ≥1.30 threshold has primarily been supported in other sports such as football and volleyball, and its transferability to handball requires empirical validation. Handball presents distinct injury patterns, including a high prevalence of shoulder overuse complaints related to repetitive throwing, as well as contact-related ankle and knee injuries. These sport-specific demands may not be fully captured by lower-limb dominated external-load indicators, and league-specific match density and recovery practices may further shape week-to-week load dynamics. Therefore, the present findings should be interpreted as associative and hypothesis-generating, motivating future handball-specific work that links cluster-derived high-load exposure and ACWR dynamics to prospectively recorded injury incidence and time-loss outcomes.

### Limitations and Future Research

Internal loads and readiness were not measured. Internal-load and readiness markers (e.g., heart-rate metrics, session-RPE, HRV, sleep or wellness indices) were not available in this dataset, limiting appraisal of individual physiological and psychophysiological responses. Consequently, the same external-load exposure may have represented different levels of internal strain depending on contextual factors such as fatigue, stress, sleep, or recovery status. Accordingly, the identified clusters should be interpreted as external-load profiles rather than comprehensive load constructs. Future studies should integrate external-load clustering with concurrent internal-load and wellbeing monitoring (e.g., session-RPE, HRV such as lnRMSSD, and brief wellness instruments) to improve interpretability and applied relevance.

*Sample scope*: data were obtained from a single elite men’s team and cover only the first four months of the season. Studies across different age groups, sexes and competition levels are required for broader generalization. Given that spatial offensive performance characteristics can differ between men’s and women’s elite competitions (Gómez-López et al., 2024), future studies should also test whether cluster-derived high-load patterns and ACWR dynamics generalize to women’s handball.

*Injury outcomes not available*: weeks with ACWR spikes were not linked to prospectively recorded injury events; therefore, the ACWR ≥1.30 should be interpreted as a risk proxy rather than an injury outcome in this dataset. Because the threshold evidence base is mainly derived from other sports, and because handball features distinct injury profiles (e.g., throwing-related shoulder overuse) and potential league-specific scheduling and recovery practices, future studies should prospectively track injury incidence in relation to ACWR spikes and high-load clustering to establish handball- and position-specific thresholds.

*Algorithm choice*: k-means depends on specific methodological choices (cluster number, z-scoring). Alternative algorithms (hierarchical, DBSCAN) may reveal different patterns. Despite these constraints, the study is one of the few to apply multivariate load tracking and position specific analytics for early warning in handball, offering a baseline for future research. Moreover, the modest silhouette values (Table S1) indicate moderate cluster overlap; therefore, findings should be validated in larger samples and, where possible, with alternative clustering approaches (e.g., hierarchical clustering or density-based methods).

Future handball-specific monitoring frameworks should integrate external-load clustering with concurrent internal-load and recovery/readiness measures, such as session-RPE, HRV (e.g., lnRMSSD), and brief wellness instruments (e.g., the Hooper Index). This would allow verification of whether ‘high-load’ clusters correspond to sessions perceived as high strain, and whether post-match recovery practices (e.g., cold-water immersion, sleep-extension strategies) differ by playing position and modulate week-to-week ACWR dynamics.

## Conclusions

Using data-driven external-load analytics, this study showed that (i) matches imposed higher mechanical demand than training sessions, (ii) wing and centre-back positions systematically accrued the greatest load, and (iii) weeks with the ACWR ≥1.30 could be predicted when ≥15% of sessions in the preceding week fell into the high-load cluster. Tracking position-specific load and combining ACWR with high-load-session density appear critical for sustaining performance while mitigating injury risk. Longitudinal work integrating internal-load indicators and alternative clustering algorithms would further strengthen data-informed load-management protocols in handball.
